# Age-dependent upregulation of Y RNAs in *Caenorhabditis elegans*

**DOI:** 10.17912/micropub.biology.000452

**Published:** 2021-09-27

**Authors:** Sangsoon Park, Jooyeon Sohn, Sujeong Kwon, Eun Ji E Kim, Yoonji Jung, Hae-Eun H. Park, Sieun S. Kim, Seung-Jae V. Lee

**Affiliations:** 1 Department of Biological Sciences, Korea Advanced Institute of Science and Technology, Daejeon 34141, South Korea.

## Abstract

Y RNA is a conserved small non-coding RNA whose functions in aging remain unknown. Here, we sought to determine the role of *C. elegans* Y RNA homologs, CeY RNA (CeY) and stem-bulge RNAs (sbRNAs), in aging. We found that the levels of CeY and sbRNAs generally increased during aging. We showed that CeY was downregulated by oxidative and thermal stresses, whereas several sbRNAs were upregulated by oxidative stress. We did not observe lifespan phenotypes by mutations in CeY-coding *yrn-1*. Future research under various genetic and environmental conditions is required to further evaluate the role of Y RNA in *C. elegans* aging.

**Figure 1:**
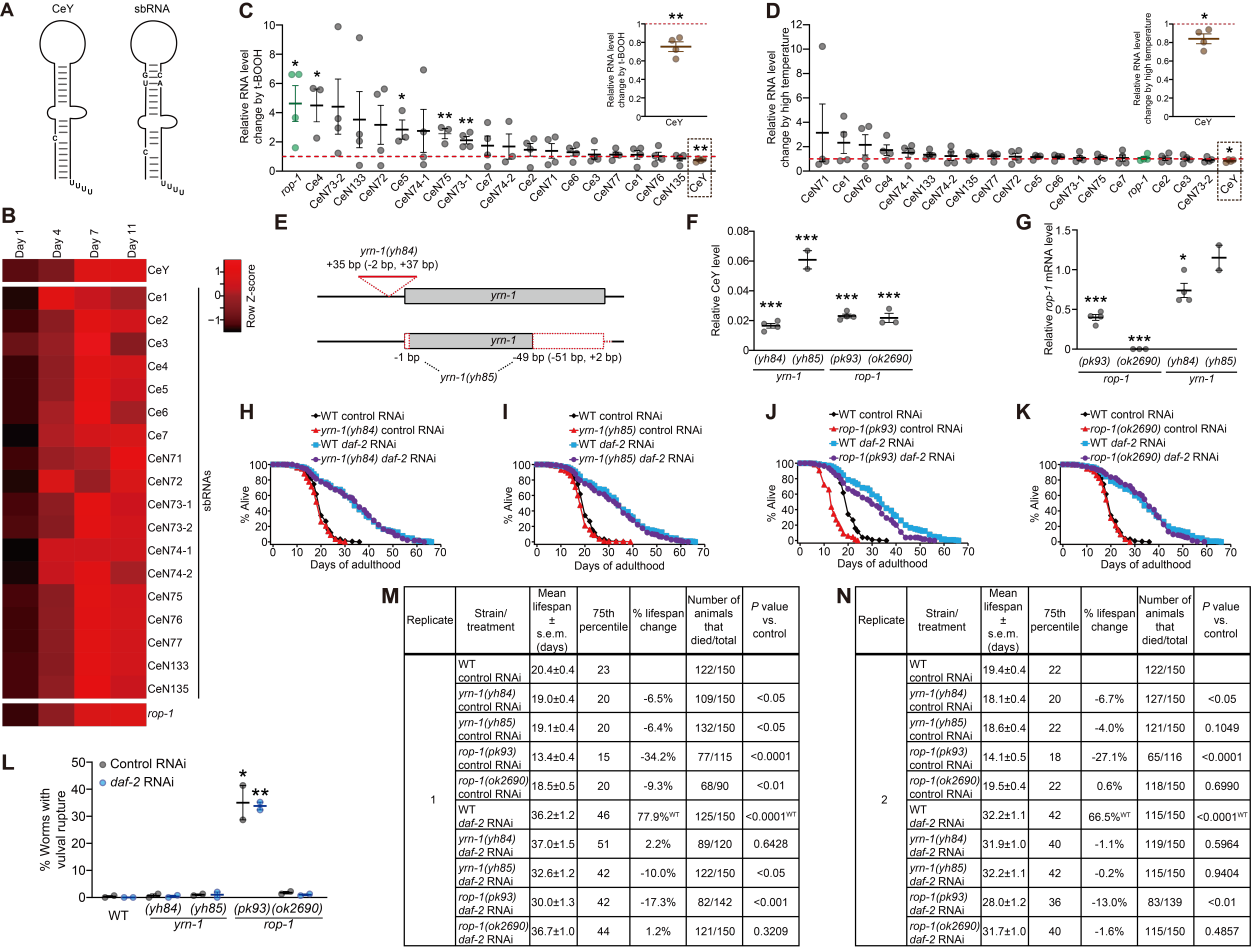
**Age-dependent changes in *C. elegans* Y RNA homologs, and the effects of *yrn-1* and *rop-1* mutations on lifespan.** (**A**)A schematic showing the conserved structure of *C. elegans* Y RNA homologs, CeY RNA (CeY) and stem-bulge RNAs (sbRNAs). Each of CeY and sbRNAs contains a conserved cytidine bulge (Van Horn *et al*. 1995; Deng *et al*. 2006; Boria *et al*. 2010), a characteristic of Y RNAs. Each sbRNA also contains a highly conserved UG-CA motif. (**B**) A heat map showing fold changes in the levels of the CeY, sbRNAs, and *rop-1* mRNA in wild-type *C. elegans* at days 4, 7, and 11 of adulthoods compared to those in day 1 animals, which were analyzed using qRT-PCR [n=3 except for CeY (n=6), *rop-1* (n=5), CeN71 (n=5), and CeN72 (n=5)]. (**C** and **D**) qRT-PCR analysis for changes in the levels of CeY (tan), sbRNAs (gray), and *rop-1* mRNA (green) under oxidative stress [7.5 mM tert-butyl hydroperoxide (t-BOOH)] [(**C**), n=4 except for CeN74-2 (n=3), CeN75 (n=3), Ce4 (n=3), and Ce5 (n=3)] and thermal stress (33°C) conditions [(**D**), n=4]. Red dotted lines indicate relative RNA levels in control conditions, which are normalized to one. Inset scatter plots show relative RNA levels of CeY (dotted box). [We noticed that a part of our data in panel **D** are not consistent with a previous report (Deng *et al*. 2006). By using northern blot analysis, Deng *et al*. reported that the levels of several sbRNAs, including CeN71, CeN72, CeN73-2, CeN74-1, CeN76, and CeN77, were increased with heat shock (30°C) for 3 hours. In contrast, we treated worms with 33°C for 30 minutes, which may have caused the difference.] n: the number of biological repeats. *P* values were calculated by using two-tailed Student’s *t* test (*P < 0.05, **P < 0.01). (**E**) A schematic showing the regions altered by *yrn-1(yh84)* and *yrn-1(yh85)* mutant alleles generated by CRISPR/Cas9-mediated genome editing. *yrn-1(yh84)* caused 35 base pair (bp) indel mutation (2 bp deletion and 37 bp insertion) at 4 bp upstream of *yrn-1*. *yrn-1(yh85)* caused one bp deletion at the 5’ end of *yrn-1*, 51 bp deletion spanning 45 bp at the 3’ end and 6 bp immediately downstream of *yrn-1*, and 2 bp insertion immediately downstream of the 45 bp deletion site. See Methods for sequence information of *yrn-1(yh84)* and *yrn-1(yh85)* mutations. (**F** and **G**) qRT-PCR analysis for the effects of *yrn-1(yh84)* (n=4), *yrn-1(yh85)* (n=2), *rop-1(pk93)* (n=4), and *rop-1(ok2690)* (n=3) on the levels of CeY (**F**) and *rop-1* mRNA (**G**). Levels of CeY and *rop-1* mRNA in wild-type are normalized to one. *P* values were calculated by using two-tailed Student’s *t* test (*P < 0.05, ***P < 0.001). (**H**–**K**) Pooled lifespan curves of wild-type (WT) and *yrn-1(yh84)* (**H**), *yrn-1(yh85)* (**I**), *rop-1(pk93)* (**J**), and *rop-1(ok2690)* (**K**) mutant animals treated with control RNAi or *daf-2* RNAi. (**L**) Percent animals that displayed vulval rupture phenotypes and that were subsequently censored during lifespan assays [panels **H** to **K**, n (trial number) =2]. *P* values were calculated by using two-tailed Student’s *t* test (*P < 0.05, **P < 0.01). (**M** and **N**) Statistical analysis of individual replicates of lifespan assays (panels **H** to **K**). All percent lifespan changes and *P* values were calculated against WT animals within each control or *daf-2* RNAi-treated groups otherwise noted with superscripts. ^WT^: percent lifespan change or *P* value against WT in a control RNAi condition within replicate. *P* values were calculated using Mantel-Cox log-rank test. s.e.m. indicates the standard error of mean.

## Description

Various non-coding RNAs (ncRNAs) change their levels during aging, and influence lifespan in many species, including *Caenorhabditis elegans* (Garg and Cohen 2014; He *et al*. 2018; Kim and Lee 2019, Kim *et al*. 2021). However, the role of Y RNAs, ncRNAs that regulate DNA replication and RNA quality control (Kowalski and Krude 2015), in aging and lifespan remains unknown. *C. elegans* genome encodes 19 Y RNA homologs, *C. elegans* Y RNA (CeY) encoded by *yrn-1* and 18 stem-bulge RNAs (sbRNAs), each of which contains a conserved stem-loop structure (Fig. 1A) (Kowalski and Krude 2015). Y RNAs form a Ro60 ribonucleoprotein by binding Ro 60-kDa protein (Ro60), which is conserved from bacteria to human (Sim *et al.* 2020). ROP-1, the *C. elegans* Ro60, binds and stabilizes CeY (Van Horn *et al*. 1995; Labbé *et al*. 1999). *rop-1* mRNA and ROP-1 protein levels increase during aging in *C. elegans* (Liang *et al*. 2014; Mansfeld *et al*. 2015; Angeles-Albores *et al*. 2017), suggesting the potential aging-regulatory functions of ROP-1 and its associated RNA, CeY. Here, we sought to determine the role of the Y RNA homologs in aging and lifespan.

We first tested whether the levels of *C. elegans* Y RNA homologs changed during aging by performing real-time quantitative reverse transcription PCR (qRT-PCR) analysis. We found that the levels of the CeY and sbRNAs were generally increased during aging (Fig. 1B). In addition, we showed that the mRNA level of *rop-1* was increased during aging (Fig. 1B), consistent with previous reports (Mansfeld *et al.* 2015; Angeles-Albores *et al*. 2017).

Increased stress resistance is associated with longevity in multiple species (Epel and Lithgow 2014, Park *et al*. 2017), and stress-responsive factors play crucial roles in aging (Son *et al*. 2019). We therefore measured the effect of oxidative or thermal stresses on the levels of *C. elegans* Y RNA homologs. We found that the level of CeY was decreased under both oxidative (Fig. 1C) and thermal stress conditions (Fig. 1D).We also showed that oxidative stress significantly increased the levels of four sbRNAs, Ce4, Ce5, CeN75, and CeN73-1 (Fig. 1C), whereas thermal stress did not (Fig. 1D; see Figure 1D legend for discussion). In contrast to the decrease in the level of CeY under these tested stress conditions, the mRNA level of *rop-1* was increased by oxidative stress (Fig. 1C) but not by thermal stress (Fig. 1D). Thus, it seems likely that these two external stresses affect the expression of various *C. elegans* Y RNA homologs and *rop-1* differently.

We then focused our functional analysis on CeY by using CRISPR/Cas9 gene targeting, because the potential functional redundancy of 18 sbRNAs may hinder characterizing the roles of individual sbRNAs in aging. We generated two mutant alleles, *yh84* and *yh85*, in *yrn-1* gene that encodes CeY (Fig. 1E). We showed that *yh84* or *yh85* mutation substantially reduced the level of CeY (Fig. 1F). In addition to these *yrn-1* mutants, we characterized two different *rop-1* mutant alleles, *rop-1(pk93)* and *rop-1(ok2690)*, both of which substantially reduced the level of CeY and *rop-1* mRNA (Fig. 1F, G) (Labbé *et al*. 1999). We then measured the lifespan of these mutants, and found that *yrn-1(yh84)* or *yrn-1(yh85)* mutation did not affect the lifespan of wild-type animals at 20°C (Fig. 1H, I, M, N). Reducing insulin/IGF-1 signaling via inhibition of *daf-2*/insulin/IGF-1 receptor doubles lifespan (Kenyon *et al*. 1993), and is one of the most studied longevity-promoting interventions in *C. elegans* (Altintas *et al*. 2016). A major function of insulin/IGF-1 signaling in *C. elegans* is to regulate the proper formation of dauer, an alternative hibernation-like developmental stage (Hu 2007). *rop-1(pk93)* mutations decrease dauer formation in a wild-type background, and enhance or suppress the increased dauer formation by *daf-2(-)* mutants in an allele-specific manner (Labbé *et al*. 2000). These findings raise the possibility that *rop-1* and/or CeY affects physiological processes regulated by insulin/IGF-1 signaling. However, *yrn-1(yh84)* or *yrn-1(yh85)* mutation had little effect on the lifespan of *daf-2* RNAi-treated worms (Fig. 1H, I, M, N). Therefore, CeY appears to be dispensable for maintaining normal lifespan and promoting longevity by reduced insulin/IGF-1 signaling. We also found that *rop-1(pk93)* mutations decreased the lifespan of both control RNAi- and *daf-2* RNAi-treated worms (Fig. 1J, M, N), whereas *rop-1(ok2690)* mutation did not (Fig. 1K, M, N). A previous study reported that *rop-1(pk93)* mutations do not affect lifespan (Labbé *et al*. 1999). We found that *rop-1(pk93)* mutations substantially increased vulval rupturing during adulthood (Fig. 1L), which reduces lifespan (Leiser *et al*. 2016). Thus, mutations in *rop-1* do not appear to directly affect aging *per se* but may cause early deaths during adulthood by increasing vulval rupture phenotypes in our experimental conditions. Together, we did not find apparent lifespan phenotypes in *yrn-1* or *rop-1* mutants, both of which displayed substantially reduced CeY levels.

Our study points to the possibility that CeY may not directly participate in the regulation of lifespan*.* CeY alone may not be functionally equivalent to the vertebrate Y RNA, as CeY does not affect DNA replication, likely because of the lack of conserved motif in the upper stem (Gardiner *et al*. 2009; Boria *et al*. 2010; Kowalski *et al*. 2015). In contrast, sbRNAs contain a conserved UG-CA motif (Fig. 1A) and participate in DNA replication (Kowalski *et al.* 2015). Thus, it will be important to test whether the inhibition of sbRNAs affects lifespan. In particular, investigating the roles of four oxidative stress-induced sbRNAs, Ce4, Ce5, CeN75, and CeN73-1, which were also age-dependently upregulated, in aging and lifespan will be crucial for future research.

## Methods

**qRT-PCR analysis.** qRT-PCR analysis was performed as previously described (Park *et al*. 2020). For qRT-PCR analysis using aged worms, wild-type (N2) animals were synchronized at L1 stage by embryo bleaching (Stiernagle 2006) followed by incubation in S-basal medium at 20°C with a gentle rotation for 16 h. Approximately 4,800 to 9,000 synchronized L1 animals were placed and cultured on nematode growth medium (NGM) plates seeded with *E. coli* OP50. L4 larvae were then treated with 50 µM 5-fluoro-2′-deoxyuridine (FUDR; Sigma-Aldrich, MO, USA) to prevent progeny from hatching. Fertile adult animals at days 1, 4, 7, or 11 were harvested and used for total RNA extraction. Total RNA was extracted by using RNAiso plus (Takara, Shiga, Japan), and cDNA was synthesized by using ImProm-II Reverse Transcriptase (Promega, WI, USA) following manufacturer’s instruction. Real-time quantitative PCR was performed using *Power* SYBR Green PCR master mix (Applied Biosystems, Thermo Fisher Scientific, Waltham, MA, USA) and StepOne real-time PCR system (Applied Biosystems, Thermo Fisher Scientific, Waltham, MA, USA). Relative quantity was calculated by comparative *C*_T_ method using *pmp-3* and small nucleolar RNA sn2343 as endogenous controls for *rop-1* mRNA and CeY/sbRNAs, respectively. *P* values were calculated by two-tailed Student’s *t* test. For qRT-PCR analysis using animals under oxidative or thermal stress conditions, approximately 5,600 stage-synchronized wild-type animals were grown on NGM plates seeded with OP50 at 20°C until they reached L4 stage. The L4 larvae were then exposed to 7.5 mM tert-butyl hydroperoxide (t-BOOH) for 4 h for eliciting oxidative stress (Goh *et al*. 2018) or placed in a 33°C incubator for 30 min for thermal stress (Brunquell *et al*. 2016). The animals were then harvested and washed three times with M9 buffer, and stored at -80°C before total RNA extraction and qRT-PCR analysis. A heat map showing age-dependent changes in levels of Y RNAs and *rop-1* mRNA was generated by Heatmapper (http://www.heatmapper.ca) (Babicki *et al*. 2016). For qRT-PCR analysis of CeY or *rop-1* mRNA using *yrn-1* (IJ1991 and IJ1992) and *rop-1* mutants (MQ470 and RB2032), approximately 6,000 to 8,000 bleach-synchronized animals were grown until developing to prefertile adults. The animals were then harvested and washed three times with M9 buffer, and stored at -80°C before extracting total RNA for qRT-PCR analysis.

**CRISPR/Cas9 genome editing.***yrn-1* mutants were generated by using CRISPR/Cas9 genome editing methods as previously described with modifications (Ghanta and Mello 2020). Day 1 adult N2 animals were injected with mixture of Alt-R CRISPR-Cas9 tracrRNA [15 µM; Integrated DNA Technologies (IDT), Coralville, IA, USA], Alt-R CRISPR-Cas9 crRNAs [*dpy-10* crRNA and two different *yrn-1* crRNAs (5 µM each); IDT], and Alt-R *S. pyogenes* HiFi Cas9 Nuclease V3 (0.5 µg/µl; IDT). Individual animals were then placed on different plates, and allowed to lay eggs. F_1_ progeny that displayed dumpy and/or roller phenotypes were individually transferred to new plates and subsequently genotyped using PCR to identify *yrn-1* mutations. *yrn-1* mutations were confirmed with Sanger sequencing (Solgent, Daejeon, South Korea). The sequences of the mutant alleles generated here are listed below. Capital letters indicate *yrn-1*, and lowercase letters indicate upstream and downstream sequences of *yrn-1*.

Wild-type: ttaaacatttGGGCTCGGTCCGAGTTTCATGGTCTCCAATGTGTGTGTGTGTGTGTTTTCTTTAGGAACC TCGGTTCCAACCTCATCTTGACCTTGAAACTACTTTGACCGCTCCttttggattt

*yrn-1(yh84)*: ttaaataggagaaataagacactaagacaataagagaaatattttGGGCTCGGTCCGAGTTTCATGGTCT

CCAATGTGTGTGTGTGTGTGTTTTCTTTAGGAACCTCGGTTCCAACCTCATCTTGACCTTGAAACTACTT

TGACCGCTCCttttggattt

*yrn-1(yh85)*: ttaaacatttGGCTCGGTCCGAGTTTCATGGTCTCCAATGTGTGTGTGTGTGTGTTTTCTTTAGGAACC

ccattt

**Lifespan analysis.** Lifespan assays were performed as previously described with some modifications (Park *et al.* 2021). Plates for feeding RNAi induction were prepared by growing HT115 RNAi bacteria containing empty vector (pAD12; control RNAi) or *daf-2* double-stranded RNA-expressing vector (pAD48; *daf-2* RNAi) on NGM plates containing ampicillin (50 μg/ml; USB, Santa Clara, CA, USA) and isopropyl-β-D-thiogalactoside (1 mM; Gold Biotechnology, St. Louis, MO, USA) at 37°C. Stage-synchronized wild-type, outcrossed *yrn-1(yh84)* and *yrn-1(yh85)* mutants, and *rop-1(pk93)* and *rop-1(ok2690)* mutants were placed on the plates and fed with control or *daf-2* RNAi bacteria at 20°C until reaching a prefertile adult stage. The animals were then transferred to new plates containing 5 µM FUDR. Animals that did not respond to a gentle touch using a platinum wire were counted as dead. Animals that ruptured, bagged, crawled off or burrowed the plates were censored but included in the statistical analysis. Lifespan assays were performed as duplicates by two independent researchers double-blindly. The lifespan data from two replicates were pooled to generate lifespan curves shown in Fig. 1H-K, but the statistical analysis was performed separately and shown in Fig. 1M and 1N. Statistical analysis of lifespan data was performed using OASIS2 (http://sbi.postech.ac.kr/oasis2) (Han *et al.* 2016), and *P* values were calculated by Log-rank (Mantel-Cox) test.

## Reagents

Following *C. elegans* strains were used in this study: wild-type Bristol N2, IJ1991 *yrn-1(yh84) IV*, IJ1992 *yrn-1(yh85) IV*, IJ1832 *yrn-1(yh84) IV* (IJ1991 outcrossed 4 times to Lee laboratory N2), IJ1851 *yrn-1(yh85) IV* (IJ1992 outcrossed 4 times to Lee laboratory N2), MQ470 *rop-1(pk93) V*, RB2032 *rop-1(ok2690) V*, IJ1876 *rop-1(pk93) V* (MQ470 outcrossed 4 times to Lee laboratory N2), IJ1887 *rop-1(ok2690) V* (RB2032 outcrossed 4 times to Lee laboratory N2). MQ470 and RB2032 were provided by CGC. HT115 RNAi bacteria containing pAD12 and pAD48, gifts from Cynthia Kenyon (Addgene plasmid # 34832 and #34834), were used as control RNAi and *daf-2* RNAi bacteria, respectively (Dillin *et al*. 2002).

Following Alt-R CRISPR-Cas9 crRNAs were obtained from Integrated DNA Technologies (IDT, Coralville, IA, USA) and used for generating *yrn-1(yh84)* and *yrn-1(yh85)* mutants:*dpy-10* crRNA (Ce.Cas9.DPY-10.1.AQ): GCUACCAUAGGCACCACGAGGUUUUAGAGCUAUGCU*yrn-1* crRNA-5’ #1: UUAGUUUAUGUUUAAACAUUGUUUUAGAGCUAUGCU (for generating *yh84*)*yrn-1* crRNA-3’ #1: ACUACUUUGACCGCUCCUUUGUUUUAGAGCUAUGCU (for generating *yh84*)*yrn-1* crRNA-5’ #2: UAUGUUUAAACAUUUGGGCUGUUUUAGAGCUAUGCU (for generating *yh85*)*yrn-1* crRNA-3’ #2: ACUCGGAAAUCCAAAAGGAGGUUUUAGAGCUAUGCU (for generating *yh85*)

Following primers were used for qRT-PCR analysis:*pmp-3* forward: GTTCCCGTGTTCATCACTCAT*pmp-3* reverse: ACACCGTCGAGAAGCTGTAGA sn2343 forward: CGGCTGTGATGATTTCCTATTG sn2343 reverse: CGGCTCAGCCTTTTCCAAG CeN71 forward: GAATTCCTGCGGTCCGGATC CeN71 reverse: AATTCCGGTAGTCAAGTTAG CeN72 forward: ATCATCGGTCCGGTGTTGATG CeN72 reverse: AACATCGGTCAAATTTGTGATGATG CeN73-1 forward: GGAGTTGATGGGTTACCAGATTA CeN73-1 reverse: GGTGTCGATGGGAAGaGG CeN73-2 forward: AGTTGATGGGTTACCCAGTC CeN73-2 reverse: GGTGTCGATGGGAAGtGT CeN74-1 forward: GGCGTCAGTGGGTTATCGTA CeN74-1 reverse: ACGCAACGGTCAAGTcGA CeN74-2 forward: TCTAAAATTAAATATATTATTCTTTTATAACGTCAGTGGGTTATCAAG CeN74-2 reverse: AGCAACGGTCAAGTgGG CeN75 forward: TACGGTCCGGAGTCGGTG CeN75 reverse: TTTTCATCGGTCAAGTTGGTGTC CeN76 forward: AGACAGGCGTGGTCCGG CeN76 reverse: CAGGAACGGTCAAGTTGGTG CeN77 forward: AATTCGGTCCGGAGTCAATG CeN77 reverse: AACGCCGGTCAAGTTGTTG CeN133 forward: ATCGGTCCGAAGTTGATGGG CeN133 reverse: TTCGATCAAGTTGATGTTGATGG CeN135 forward: ATCAGGCATAGGTCCGGAG CeN135 reverse: AACAGGAAGTCAAGTTGTTGCC Ce1 forward: CGACCACCGGTCCGGAG Ce1 reverse: ACCCCCGGTCAAGGGATG Ce2 forward: TGGCCGTCCGGGTCTG Ce2 reverse: ACCTTCCCGGTCAAGGAG Ce3 forward: GTCCGGAGATTATGGGTTATC Ce3 reverse: TATGAAGAACACGGAGAGATTAAC Ce4 forward: AGTTCGGTCCGGAGTTGATG Ce4 reverse: CGGTCAAGTTGTTGTTGATGG Ce5 forward: AGTTCGGTTCGGAGTTGATG Ce5 reverse: GGTCAAGTTGTTGTTGATGGG Ce6 forward: ATCGGTCCGGCGTCAGTG Ce6 reverse: TGTAACTGTCAAGTTGATGCCG Ce7 forward: TCGGTCCGGCGCCAATAG Ce7 reverse: TATCAGTCAAGTTGGTGCCAATG*yrn-1* forward: TCGGTCCGAGTTTCATGGTC*yrn-1* reverse #1: AGCGGTCAAAGTAGTTTCAAGGT (for Fig. 1B-D)*yrn-1* reverse #2: GTTCCTAAAGAAAACACACACAC (for Fig. 1F)*rop-1* forward #1: ATGAGACTCTCTAACGTCTGC (for Fig. 1B-D)*rop-1* reverse #1 GACTTTCTCCATTTGACGAGG (for Fig. 1B-D)*rop-1* forward #2: ACTACGTTAACAACCTTGATTTCG (for Fig. 1G)*rop-1* reverse #2: CAGCCCAGGTATCGTTGTC (for Fig. 1G)
